# The student-institution fit at university: interactive effects of academic competition and social class on achievement goals

**DOI:** 10.3389/fpsyg.2015.00769

**Published:** 2015-06-15

**Authors:** Nicolas Sommet, Alain Quiamzade, Mickaël Jury, Gabriel Mugny

**Affiliations:** ^1^Unité de Psychologie Sociale, Faculté de Psychologie et des Sciences de l’Education, Université de Genève, Genève, Switzerland; ^2^UnilaPS, Institut de Psychologie, University of Lausanne, Switzerland; ^3^Distance Learning University, Sierre, Switzerland; ^4^Laboratoire de Psychologie Sociale et Cognitive, Centre National de la Recherche Scientifique, UMR 6024, Université Clermont Auvergne, Clermont-Ferrand, France

**Keywords:** academic competition, social class, first- and continuing-generation students, achievement goals, student-institution fit, achievement gap

## Abstract

As compared to continuing-generation students, first-generation students are struggling more at university. In the present article, we question the unconditional nature of such a phenomenon and argue that it depends on structural competition. Indeed, most academic departments use harsh selection procedure all throughout the curriculum, fostering between-student competition. In these departments, first-generation students tend to suffer from a lack of student-institution fit, that is, inconsistencies with the competitive institution’s culture, practices, and identity. However, one might contend that in less competitive academic departments continuing-generation students might be the ones experiencing a lack of fit. Using a cross-sectional design, we investigated the consequences of such a context- and category-dependent lack of fit on the endorsement of scholastically adaptive goals. We surveyed *N* = 378 first- and continuing-generation students from either a more competitive or a less competitive department in their first or final year of bachelor’s study. In the more competitive department, first-to-third year decrease of mastery goals (i.e., the desire to learn) was found to be steeper for first- than for continuing-generation students. In the less competitive department, the reversed pattern was found. Moreover, first-to-third year decrease of performance goals (i.e., the desire to outperform others) was found to be steeper within the less competitive department but did not depend on social class. This single-site preliminary research highlights the need to take the academic context into account when studying the social class graduation gap.

## Introduction

In Western culture, higher education institutions ideally aim at ensuring equality of opportunities, that is, selecting impartially the more competent students, independently of their social class. However, they ironically tend to reproduce social inequalities, selecting preferentially the higher-class students (for a review, see Aronson, 2008). As a matter of fact, in comparison with continuing-generation students (for whom at least one parent has a college degree, i.e., the middle/upper class), first-generation students (for whom neither parent has a college degree, i.e., the lower class) are 68% less likely to earn a college degree after 4 years of college (DeAngelo et al., 2011). This social class graduation gap has been documented in European Union (OECD, 2014; see indicator A4, pp. 84–85) as well as in Switzerland (SKBF, 2014; see pp. 178–179). It is notably explained by a lack of fit between the values of first-generation students and that promoted by universities (for a review, see Stephens et al., 2014b).

Most often, scholars seem to consider first-generation students as unconditionally disadvantaged, and continuing-generation as privileged in the educational system (for a review, see Spiegler and Bednarek, 2013). Yet, one might argue that they only look at one side of the coin. Reasoning in terms of person-environment fit (e.g., Holland, 1959), the degree of one’s sense of fit may indeed differ according to the context. For instance, although lower-class students have a lower likelihood to graduate from more competitive colleges, fields, or departments (Aries and Seider, 2005), some evidence suggests that higher-class students are less likely to succeed in less competitive ones (Agan, 2013; see also, Davies and Guppy, 1997; Triventi, 2013; Davies et al., 2014). Additionally, first-generation students are underrepresented in less prestigious departments (e.g., Sciences; Chen and Carroll, 2005), top-tier colleges (Carnevale and Rose, 2003), and elite institutions (Albouy and Wanecq, 2003). But conversely, continuing-generation students are underrepresented (or less represented) in less prestigious fields (e.g., vocational/technical), second-to-fourth-tier colleges, and second-rate institutions. One might argue that these findings translate differences in terms of fit as a function of both social class *and* academic competition.

With most research describing first- and continuing-generation students as—respectively—“not fitting in” and “fitting in” in absolute terms, we propose a more even-handed approach raising the possibility of a relative lack of fit. Specifically, we suggest a categorical and context-dependent lack of fit might impair the level of endorsement of two academically adaptive achievement goals, namely the desire to learn and that to perform. Within a more competitive department, first-generation students may experience a lower student-institution fit, hindering the pursuit of these goals. Contrariwise, within a less competitive department, continuing-generation students may also experience a lower fit, hindering the endorsement of these same goals.

### A Context-Independent View of Social Class and Student-Institution Fit

Let us first consider the relationship between social class and student-institution fit independently of the academic context, that is, the mere correspondence between personal and environmental characteristics (Denson and Bowman, 2014). In addition to economic (i.e., lower financial resources; Desimone, 1999) and social factors (e.g., parenting practices; Guryan et al., 2008), psychological reasons might account for the social class graduation gap. For instance, Bourdieu and Passeron (1977) argued that students from lower social class could experience a discontinuity between their *habitus* (i.e., schemes of perception, thought, and action, inherited from their socio-cultural background) and the higher-class habits promoted by universities. Such a discrepancy would result in lower achievement (for empirical evidences, see Gaddis, 2013).

More recently, Stephens et al. (2012a) specified the effects of social class on student-institution fit. On the one hand, authors showed that first-generation students regulated their behaviors in keeping with *interdependent* values. They endeavor to adjust themselves to the context, to be connected to others and to respond to others’ interests. On the other hand, authors showed that continuing-generation students regulated their behaviors in keeping with *independent* values. They try to influence the context, to be distinct from others, and to satisfy their own needs, preferences, and interests (see also Stephens et al., 2007). Yet, higher education institutions most often convey *independent* norms, according to which students are expected to work independently, to strive for personal achievement, and to express their own views (Greenfield et al., 2000). In these contexts, first-generation students therefore tend to experience a low sense of student-institution fit, whereas continuing-generation students experience a high fit. In a series of articles, Stephens and her colleagues reported that such a reduction in terms of academic fit led first-generation students to feel more stressed (i.e., higher cortisol levels; Stephens et al., 2012b), to obtain lower grades (Stephens et al., 2012a, Study 2), and to achieve lower academic success (Stephens et al., 2014a).

### A Context-Dependent View of Social Class and Student-Institution Fit

Let us now consider the variations in the relationship between social class and student-institution fit as a function of the academic context. Most of the studies showing first-generation students’ lack of academic fit were conducted in high-ranked competitive universities (see Granfield, 1991). Yet, although most higher education institutions and departments are highly competitive (only a limited number of students are allowed to proceed to the next year), some others are less competitive (see De Paola, 2011). Moreover, whereas the former promote independent values, the latter might promote different values, or—at least—less independent ones. As a matter of fact, Stephens et al. (2012a, Study 1b) reported that university administrators of highly competitive institutions (i.e., top-tier colleges) characterized the values promoted by their university as being *more independent* than the ones of mildly competitive institutions (i.e., second-tier colleges). In the first instance, we will draw on these observations and develop the idea that more vs. less competitive departments differ drastically regarding their (i) institutional culture, (ii) institutional practices, and (iii) institutional identity. Then, we will argue that the relationship between social class and student-institution fit depends on these differences.

#### Competition and Institutional Culture

As a function of academic competition, departments convey different cultures in terms of excellence and individualism. In more competitive departments, students are encouraged to develop their idiosyncrasies and critical judgment (e.g., in Medicine, Kennedy et al., 2009). As an example, Skelton (2005) urged higher education administrators to “[promote] the enhancement of the individual student’s personal character [and] the development of the individual student’s autonomy” (p. 22). Conversely, in less competitive departments, the pursuit of collective goals, rather than individual ones, is emphasized (for the effects of competition on individualistic behaviors, see Barnett and Bryan, 1974). For instance, Vansteenkiste et al. (2006) reported that Education (vs. Business) students were more oriented toward helping others than toward wealth.

#### Competition and Institutional Practices

As a function of academic competition, departments rely on practices fostering different representations of self- and other-competence. In more competitive departments, where a *numerus clausus* can be established between the first and the second year, only few of the candidates will pass their final exam (Spence, 1981). In such environment, the higher the likelihood that others are selected, the lower the chance one has to succeed (i.e., negative interdependance; for a review on social interdependence theory, see Johnson and Johnson, 2005). Students enrolled in more competitive departments therefore perceive the competence of their classmates as necessarily coming into conflict with their own competence. In other words, others’ and self-competences are viewed as negatively correlated. It is less the case for students enrolled in less competitive departments, who view others’ and self-competences as uncorrelated (Sommet et al., 2013).

#### Competition and Institutional Identity

As a function of academic competition, departments imply different changes with regard to social identity, that is, the attitudinal and behavioral adjustments to comply with new institutional norms (Emler, 2005). More competitive departments (e.g., Law, Business, Medicine) are associated with superior reputation and attractiveness than the less competitive ones (James, 2000). As a matter of fact, the more competitive a department, the higher its students’ future earnings and socio-economic status (Pascarella and Terenzini, 2005). Thus, for lower-class students, being enrolled in a more competitive department involves a larger *upward social mobility process*. This was notably found to predict psychological discomfort (Iyer et al., 2009). Conversely, for higher-class students, being enrolled in a less competitive department may involve social immobility (i.e., being just as successful as one’s parents) or *downward social mobility process* (i.e., not being as successful as one’s parents; see Stocké, 2007), which could result in status insecurity (Wilkinson and Pickett, 2008).

#### Academic Competition, Social Class, and Student-Institution Fit

What conclusion regarding social class and student-institution fit can be reached from the fact that institutional culture, practices, and identity depend on academic competition? On the one hand, in more competitive departments, first-generation students’ values of *positive interdependence* should be more incongruent with institutional *individualistic* culture and *more negatively interdependent* practices than continuing-generation students’ values. Moreover, they should experience a stronger feeling of incompatibility between their socio-familial identity, their new institutional identity, as well as with their future possible identity (i.e., more elevated status; for examples, see Reay et al., 2009, 2010; Lee and Kramer, 2013).

On the other hand, in the more specific case of less competitive departments continuing-generation students’ values of *independence* might reciprocally appear as more incongruent with institutional *collectivistic* culture and *less negatively interdependent* practices than first-generation students’ values. It is also legitimate to think that, in this case, their socio-familial identity might conflict with both their institutional identity and their future socio-economical identity (i.e., less elevated status). Such a lack of identity-related fit would occur to the extent that students perceive themselves as being engaged in a downward mobility process (Hurrelmann et al., 1988). In the present study, we will specifically focus on students’ endorsement of academically adaptive goals as a function of such a context- *and* category-dependent lack of fit.

### Student-Institution Fit and Achievement Goals Regulation

Achievement goals theorists distinguish two non-exclusive reasons for engaging in competence-relevant behaviors, namely mastery and performance goals. Mastery goals relate to the desire to personally progress, to surpass oneself, whereas performance goals pertain to the desire to relatively succeed, to surpass others (for a historical review, see Elliot, 2005). Mastery goals predict persistence after failure (Dweck and Leggett, 1988), intrinsic motivation (Rawsthorne and Elliot, 1999) and task-commitment (Poortvliet and Giebels, 2012). Performance goals predict performance, be it in experimental (Elliot et al., 2005) or field settings (Barron and Harackiewicz, 2003). In the late 90s, adopting a multiple goals perspective, Judith Harackiewicz and her colleagues (for a review, see Senko et al., 2011) showed that an elevated degree of both mastery *and* performance goals corresponded to an adaptive pattern of achievement-related behaviors. Endorsed conjointly, these goals allow the maintenance of optimal degrees in task interest (e.g., reduced intention to drop-out out from university; Fasching et al., 2011) and performance (e.g., elevated course grades; Hulleman et al., 2010).

Mastery and performance goals are not merely stable traits (Fryer and Elliot, 2007), but may also be regulated in response to environmental factors (Senko and Harackiewicz, 2005). As a matter of fact, students enter in higher education holding high mastery goals (Meier et al., 2013), but these goals tend to decline over the course of the curriculum (for a meta-analytic summary, see Corker et al., 2013). Such a decline is explained by the fact that many of the students become aware of the distance between their idealistic expectations and the reality of the courses (Lieberman and Remedios, 2007). However, performance goals tend to remain more stable, although similar discrepancy between one’s resources and task demands predicts their decline (Kumar and Jagacinski, 2011).

Hence, it does not come as a surprise that student-institution fit is predictive of the maintenance of an elevated degree of mastery and performance goals (see Eccles and Roeser, 2009). Generally speaking, the incongruence between student’s beliefs and the perception of their environment was found to deplete motivation (Byrd and Chavous, 2012), various kinds of needs (e.g., achievement; Harms et al., 2006), and the level of goals endorsement (Greguras et al., 2014). More specifically, a higher sense of match between individual preferences or values and environmental requirements or culture sustains task commitment, a mastery goal-related outcome (Blau, 1987; O’Reilly et al., 1991), as well as a high level of relative performance, a performance goal-related outcome (Goodman and Svyantek, 1999; Greguras and Diefendorff, 2009).

### Overview and Hypotheses

As suggested in the opening paragraphs, first-generation students are less likely to succeed and to be represented in more competitive academic environments, whereas continuing-generation students are less likely to succeed and to be represented in less competitive ones. It reflects the fact that first-generation students may experience a discrepancy between their and the more competitive institutions’ culture, practices, and identity. As a theoretical extension, the same might be true for continuing-generation students in less competitive departments. In the present article, we argue that this relative lack of student-institution fit as a function of social class *and* competition should predict the decrease in the endorsement of mastery and performance goals. We therefore formulate two hypotheses. In a more competitive department, first-generation students should report lower mastery (hypothesis 1a) and performance (hypothesis 2a) goals in the third than in the first year; it should not be the case for continuing-generation students. Conversely, in a less competitive department, continuing-generation students should report lower mastery (hypothesis 1b) and performance goals (hypothesis 2b) in the third than in the first year; it should not be the case for first-generation students.

## Materials and Methods

The study used a 2 (less vs. more competitive department) × 2 (lower vs. higher social class) × 2 (first vs. third academic year) cross-sectional design. First, the sample included undergraduates from a more and a less competitive department. In the former, namely Life Sciences, the first-to-second year passage appears to be more selective; in the latter, namely Civil Engineering, the selection is weaker (see Pilot Study). Second, first-generation students were distinguished from continuing-generation students. Lower social class students were those having no college-graduated parent, whereas higher social class students were those having at least one college-graduated parent (for a similar operationalization, see Stephens et al., 2012b). Finally, both first- and final-year Bachelor’s degree students were surveyed in order to observe the evolution of their achievement goals. The questionnaire assessed both mastery and performance goals, that is, both the will to learn and to outperform others.

### Participants and Procedure

Three hundred and eighty-eight undergraduates from a French-speaking Swiss university (EPFL, that is, the Swiss Federal Institute of Technology in Lausanne) filled in a paper-and-pencil questionnaire presented as a research on “the motivational profile of students.” Ten observations were excluded due to missing values. The final sample was composed of *N* = 378 students, 153 females and 222 males (three missing values), with a mean age of 20.01 years (*SD* = 1.72).

Academic competition was operationalized through a difference in the selection process between two departments of the university: Civil Engineering (*n* = 179) and Life Sciences (*n* = 199). Such a difference is both objective (i.e., average success rate) and subjective (i.e., perception). First, the two departments vary in terms of examination passing rates: The first-to-second year average success rate for the five academic years preceding the study was more than half for Civil Engineering (*M* = 58.51%, *SD* = 4.41%), whereas it was less than half for Life Sciences (*M* = 44.01%, *SD* = 4.99%)^1^. As compared to the average success rate of the whole EPFL (*M* = 50.53%; *SD* = 0.84%), that of Civil Engineering was higher, indicating a less competitive environment, and that of Life Sciences was lower, indicating a more competitive environment. Second, a Pilot Study aimed at confirming that students perceived Life Sciences as being more competitive than Civil Engineering. Sixty-one second-year undergraduates, mainly students of other departments but from the same institution as that of the main study, were surveyed. Seven missing observations and two outliers (|SDR| > 3.44^2^) were excluded from the analyses. The final sample comprised *N* = 52 students (i.e., four from Civil Engineering, four from Life Sciences, and 44 others), 23 women and 29 men (*M*_Age_ = 20.49, *SD* = 1.66). On a scale ranging from 1 (“not at all”) to 7 (“completely”), participants were asked to evaluate the extent to which Civil Engineering was a selective department, promoted between-student competition, and enrolled competitive students. The same three questions were repeated for Life Sciences. The order between the two sets of items was counterbalanced. The two scales showed a satisfactory reliability (αs > 0.70). Regression analyses tested the difference in terms of perceived competition between Civil Engineering and Life Sciences. Participants’ academic affiliation as well as order of item presentation were statistically controlled. As expected, results revealed that the two departments were perceived as differently competitive, *B* = 0.56, *SE* = 0.22, *F*(1, 48) = 6.45, *p* = 0.014, ${\text{η}}_{p}^{2}$ = 0.12. Life Sciences were judged as being more competitive (*M* = 4.61, *SE* = 0.26) than Civil Engineering (*M* = 4.05, *SE* = 0.20). In other words, the two departments were objectively and subjectively perceived by students of the EPFL as different in terms of competition.

Change in achievement goals was appraised using a cross-sectional design; we surveyed both first-year (*n* = 279) and third-year students (*n* = 99). As mere social class was not found to significantly predict freshmen’s mastery and performance goals (Jury et al., 2015a, Studies 1–3), students having just entered university constituted a control group. As identifying, interpreting, and responding to lack of student-institution fit are long-term processes (Caldwell et al., 2004), students in their final year before bachelor’s degree graduation constituted the group in which changes were expected. Data were collected in agreement with the Swiss Psychological Society’s ethical guidelines^3^. No experimental manipulation was performed. No incentive (nor credits neither money) was given for participation. Participants were informed that the questionnaire was anonymous and that they could refuse to do it and withdraw from participation at any time.

### Variables

#### Social Class

Participants reported the highest educational level attained by their parents using Genoud’s (2011) seven-choice scale^4^. As in prior research (e.g., Somers et al., 2004), participants were categorized as first-generation students when neither of their parents had a college degree (*n* = 101) and as continuing-generation students when at least one of their parents had a college degree (*n* = 277). Table 1 shows the number of participants as a function of the three independent variables considered.

**TABLE 1:** **Number of participants as a function of the department, the academic year and the social class**.

****	**Life Sciences (more competitive)**		**Civil Engineering (less competitive)**	
	**First year**	**Third year**	**First year**	**Third year**
First-generation	36	18	31	16
Continuing-generation	103	42	109	23

#### Achievement Goals

Participants reported their goals using the French validation of Elliot and McGregor’s (2001) Achievement Goal Questionnaire (Darnon and Butera, 2005) on a scale ranging from 1 (“not at all”) to 7 (“completely”). Three items measured their mastery-approach goals (e.g., “I want to learn as much as possible from the classes”) and three others performance-approach goals (e.g., “It is important for me to do better than other students”). A summary of descriptive statistics and correlations is presented in Table 2.

**TABLE 2:** **Cronbach’s alpha, mean, standard deviation, and correlation among study variables**.

****	**α**	***M***	***SD***	**Correlations**		
				**1**	**2**	**3**
1. Social class	n/a	n/a	n/a	—		
2. Mastery goals	0.78	5.08	1.18	0.01	—	
3. Performance goals	0.90	3.54	1.61	0.08	0.26*	—

*p < 0.01.

## Results

### Overview of the Regression Analyses

Multiple linear regression analyses were conducted with the department (coded “–0.5” for less competitive, i.e., Civil Engineering, and “+0.5” for more competitive, i.e., Life Sciences), the academic year (coded “–0.5” for first-year students and “+0.5” for third-year ones), as well as the social class (coded “–0.5” for first-generation students and “+0.5” for continuing-generation ones) as independent variables, with mastery and performance goals as dependent variables.

Complete analyses of covariance was conducted in preliminary stage (Yzerbyt et al., 2004), with gender (coded “–0.5” for women and “+0.5” for men) and mean-centered age. As including these terms did not produce significant effects on any of the outcome variables, they were not retained in the analyses. The final model contained seven predictors: the department, the academic year, the social class and all interactions. A summary of the results is displayed in Table 3.

**TABLE 3:** **Regression coefficients for the models testing the effects of department, academic year, and social class on mastery and performance goals**.

****	**Mastery goals**			**Performance goals**		
****	***B***	***SE***	${\text{η}}_{p}^{2}$	***B***	***SE***	${\text{η}}_{p}^{2}$
Intercept	4.96**	0.07	0.92	3.36**	0.10	0.75
Department	0.04	0.15	–	0.20	0.20	–
Academic year	–0.52**	0.15	0.03	–0.47*	0.20	0.01
Social class	–0.03	0.15	–	0.20	0.20	–
Department × academic year	–0.31	0.30	–	1.05*	0.41	0.02
Department × social class	0.54^†^	0.30	0.01	–0.09	0.41	–
Academic year × social class	0.03	0.30	–	0.02	0.41	–
Department × academic year × social class	1.92**	0.60	0.03	0.26	0.82	–

*p < 0.05; **p < 0.01; ^†^p < 0.1.

### Mastery Goals

**FIGURE 1:**
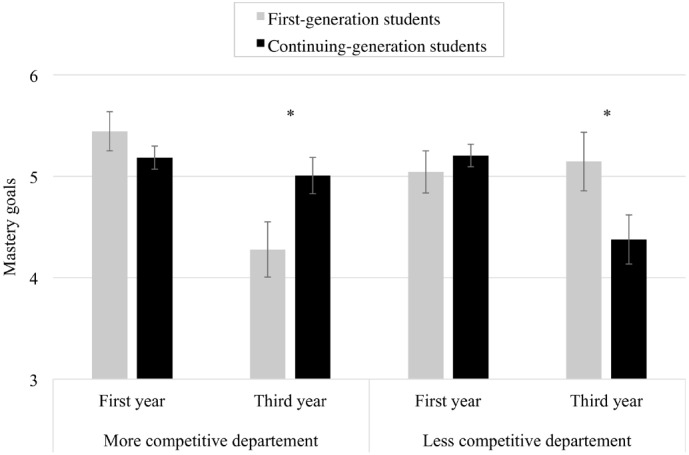
**Effects of department, academic year and social class on mastery goals.** Error bars represent standard error of the mean; asterisk (*) indicates a significant difference (*p* < 0.05) between first- and continuing-generation students.

Analyses revealed a significant interaction between the department, the academic year, and the social class on mastery goals, *B* = 1.92, *SE* = 0.60, *F*(1, 370) = 10.43, *p* = 0.01, ${\text{η}}_{p}^{2}$ = 0.03 (see Figure 1). It indicated that the interactive effects between the academic year and the social class depended on the department. This interaction was decomposed by first examining the more competitive department (hypothesis 1a) and then the less competitive one (hypothesis 1b).

First, in the more competitive department, the interaction between the academic year and the social class was significant, *B* = 0.99, *SE* = 0.40, *F*(1, 370) = 6.25, *p* = 0.013, ${\text{η}}_{p}^{2}$ = 0.02. Results confirmed that first-generation students reported lower mastery goals when in the third year than when in the first one, *B* = –1.16, *SE* = 0.33, *F*(1,370) = 12.18, *p* < 0.001, ${\text{η}}_{p}^{2}$ = 0.03. In other words, within the more competitive department, first-generation students’ mastery goals tended to decrease from the university entrance (*M* = 5.44, *SE* = 0.19) to the final year of study (*M* = 4.28, *SE* = 0.27). In line with the existing literature, these results suggest that first-generation students experience a particular discrepancy between their self and the competitive academic environment, impairing their willing to learn. Conversely, the effect of academic year was not different from 0 for continuing-generation students, *B* = –0.18, *SE* = 0.21, *F* < 1. Continuing-generation students’ mastery goals did not decrease between the first (*M* = 5.18, *SE* = 0.11) and the third year (*M* = 5.01, *SE* = 0.18).

Second, in the less competitive department, the interaction between the academic year and the social class was also significant, *B* = –0.93, *SE* = 0.44, *F*(1, 370) = 4.37, *p* = 0.037, ${\text{η}}_{p}^{2}$ = 0.01. Compared to the previous analysis, the results were reversed. Indeed, in this department, first-generation students’ mastery goals endorsement did not decrease between the first (*M* = 5.04, *SE* = 0.21) and the third year (*M* = 5.15, *SE* = 0.29), *B* = 0.10, *SE* = 0.36, *F* < 1. As first-generation students maintained an elevated degree in such a context, it conveys the idea they may not be unconditionally disadvantaged in the educational system. Conversely, continuing-generation students reported lower mastery goals when in the third year than when in the first one, *B* = –0.82, *SE* = 0.26, *F*(1,370) = 9.73, *p* = 0.002, ${\text{η}}_{p}^{2}$ = 0.03. In the less competitive department, continuing-generation students’ mastery goals tended to diminish from the university entrance (*M* = 5.20, *SE* = 0.11) to the last year (*M* = 4.38, *SE* = 0.24). This result leads into thinking that in less competitive environment continuing- rather than first-generation students are those who face the motivational consequences of a lack of student-institution fit.

Taken together, such findings sustain both ideas that first-generation students might not always have to struggle at university and that continuing-generation students might not always be favored by the academic context. Indeed, continuing-generation students could also experience a discrepancy between their self and the less competitive environment, which can deplete their desire for improvement and learning.

### Performance Goals

For performance goals, analyses did not reveal a significant second-order interaction between the department, the academic year, and the social class, *B* = –0.26, *SE* = 0.81, *F* < 1. Contrary to our second hypothesis, the interactive effects between the academic year and the social class did not depend on the department.

**FIGURE 2:**
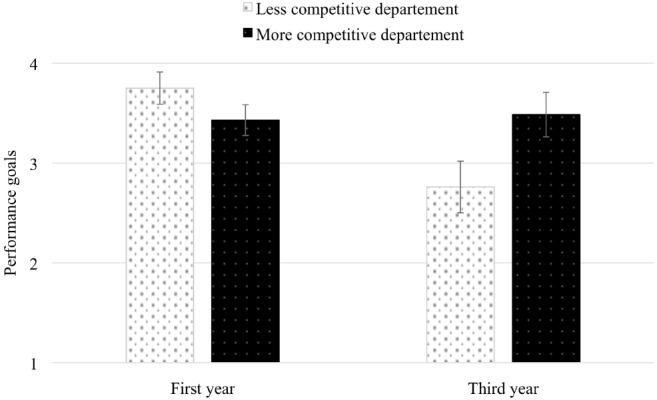
**Effects of department and academic year on performance goals.** Error bars represent standard error of the mean.

However, the first-order interaction between the department and the academic year was significant, *B* = 1.05, *SE* = 0.41, *F*(1, 370) = 6.60, *p* = 0.011, ${\text{η}}_{p}^{2}$ = 0.02. As can be seen in Figure 2, in the less competitive department, performance goals were lower in the third year than in the first one, *B* = –0.99, *SE* = 0.30, *F*(1, 370) = 10.56, *p* = 0.01, ${\text{η}}_{p}^{2}$ = 0.03. Regardless of social class, for students enrolled in the less competitive department, performance goals decreased from university entrance (*M* = 3.75, *SE* = 0.16), to the final year of study (*M* = 2.76, *SE* = 0.26). In the more competitive department, such an effect was not observed, *B* = 0.16, *SE* = 0.27, *F* < 1. Indeed, whatever the social class, performance goals did not change between the first (*M* = 3.43, *SE* = 0.15) and the third year (*M* = 3.49, *SE* = 0.22). As the endorsement of performance goals is indicative of a more competitive environment, these findings confirmed that Life Sciences were characterized by a more elevated degree of between-student competition than Civil Engineering.

## Discussion

Most research on social inequalities in higher education described first-generation students as “not fitting in” and continuing-generation ones as “fitting in,” independently of their academic environment. Such a weighed tendency might be due to two reasons. Firstly, most academic contexts are highly competitive (see Davies and Hammack, 2005), therefore specifically impairing the lack of fit of first-generation students. Secondly, most social scientists’ goals are to reduce social inequalities (see Dompnier et al., 2008, p. 250), therefore willing to bolster up the lack of fit of first-generation students. However, universities being heterogeneous in terms of culture, practices and identity (Guimond and Palmer, 1996), the student-institution fit-based approach implies that in less competitive contexts, continuing-generation students might also experience comparable self-institution discrepancies. The present study aimed at testing the effects of such a category- and context-driven lack of fit on the endorsement of two academically adaptive achievement goals (Harackiewicz et al., 2002)^5^. Congruent with our first hypothesis, in a more competitive department, first-generation students reported lower mastery goals when in third than when in first year (hypothesis 1a); it was not the case for continuing-generation students. Conversely, in a less competitive department, continuing-generation students reported lower mastery goals when in third than when in first year (hypothesis 1b); it was not the case for first-generation students. However, incongruent with our second hypothesis, such an interaction effect was not observed for performance goals. Let us see how these results contribute to connecting the literature on social inequalities and that on achievement goals, by first considering mastery goals, and then performance goals. We will then discuss some practical implications.

### Theoretical Contribution Regarding Mastery Goals

In the more competitive department, over the course of their bachelor’s study, first-generation students’ mastery goals were found to diminish to a greater extent than those of continuing-generation students. Yet, we have seen that at university mastery goals actually relate to drop-out intentions (Fasching et al., 2011). Hence, taking low mastery goals as a drop-out risk factor (for a review, see LaCombe, 2007, pp. 46–48), such a result might provide a goal-based explanation for the fact that first-generation students are much more likely to leave from more competitive institutions than continuing-generation ones (see Bowen and Bok, 1998). More generally, it might account for the lack of social class diversity in more competitive universities (Lohfink and Paulsen, 2005).

In the less competitive department, over the course of their bachelor’s study, continuing-generation students’ mastery goals were found to diminish to a greater extent than those of first-generation students. Once again, taking low mastery goals as a drop-out risk factor, such a result might provide a goal-based explanation for the disappearance of the social class attrition rate in less competitive institutions (e.g., community college, Fike and Fike, 2008). Moreover, it might explain why continuing-generation students tend to flee from less prestigious colleges (Reay et al., 2005) and to transfer to another institution when their needs are not satisfied (Herzog, 2005) or when they can benefit from an informal career opportunity requiring no given level of education (Mangino, 2012).

### Theoretical Contribution Regarding Performance Goals

As compared to the more competitive department, in the less competitive department, performance goals showed a steeper first-to-third year reduction. Generally speaking, it pertains to the fact that structural competition—in that it fosters social comparison—favors the endorsement of performance goals (Murayama and Elliot, 2012), whereas the perception of a climate not emphasizing relative performance predicts their diminution (Wolters, 2004). However, social class was not found to influence the effect of academic competition on performance goals regulation, revealing unexpected variations in the relationship between student-institution fit and achievement goals. Yet, we have seen that performance goals are related to higher academic grades (Hulleman et al., 2010). Hence, taking low performance goals as a low-grade risk factor (for a review, see LaCombe, 2007, pp. 48–50), such a null finding may echoe the inconsistent effects of social class on grades (for a review, see Spiegler and Bednarek, 2013, p. 327).

In sum, from a goal-based perspective, these findings seem to suggest that the social class graduation gap—be it context-dependent or -independent—might be explained by (i) a misfit-driven lack of learning-focus (i.e., mastery goals), *rather than* (ii) a misfit-driven lack of success-focus (i.e., performance goals). As a matter of fact, the social class graduation gap is accounted by a series of epistemic causes, namely lower interest in extracurricular activities (Terenzini et al., 1996), lower time-investment (Inman and Mayes, 1999), or lower self-efficacy (Hellman, 1996). Research should be undertaken to test the specific role of mastery goals in explaining the effects of competition and social class on drop-out and on grade.

### Practical Implications

In the last years, scholars proposed various recommendations and/or developed several interventions intended to reduce the misfit-driven social class achievement gap. Some of them are institution-focused, that is, at a macro-level, such as need-based financial aids (Destin and Oyserman, 2009). However, some others are student-focused, that is, at a micro-level, such as personal value affirmation (Harackiewicz et al., 2014). How do our results inform on the goal-related potential consequences of these two approaches?

Amongst the institution-focused approaches, as the social class achievement gap is notably attributed to “the increasing competitiveness among prospective students” (Astin and Oseguera, 2004, p. 338), some scholars urged faculty members to reduce competition (e.g., Milem et al., 1998; see also Attewell, 2001; Maroy, 2004; Alon, 2009; Smeding et al., 2013). Extending the present results, one might suspect that change in structural policies aiming at lessening competition might have ironical effect. Although reducing competition could be beneficial for the maintenance of first-generation students’ mastery goals, it could impair that of continuing-generation ones (for similar effects with gender, competition and performance, see Ors et al., 2013). In a way, Spencer and Castano’s (2007) results can be linked to this rationale. Indeed, by minimizing the evaluative dimension of a task (presenting it as non-diagnostic of intelligence), authors demonstrated that lower class students experienced less threat (see also Jury et al., 2015b), but that upper-class students experienced less challenge. Yet, the hypothesis of the potential perverse role of competition reduction on goals, in that it could undesirably impair mastery goals within the dominant group, remains to be formally tested. Before that additional empirical data confirm or infirm it, relying on student-focused approaches aimed at ensuring social equality between first- and continuing-generation students might be less hazardous. As a matter of fact, Stephens et al.’s (2014a) difference-education intervention—in which students learn about the potential consequences of social class—was found to eliminate first-generation students’ disadvantage without affecting continuing-generation students (for another example of knowledge-based intervention, see Johns et al., 2005).

### Limitations

Two limitations of the present study should be acknowledged. First, the cross-sectional design of our study does not allow to formally distinguish whether a (self-)selection process or a socialization one accounts for the results (Bachman et al., 1987). In other words, it is not possible to determine if students oriented toward mastery goals drop out when suffering from a lack of fit or if the ones suffering from a lack of fit abandon their mastery goals over time. However, as observable in Table 1, the first-to-third year diminutions of the number of first-generation students are virtually similar from one department to the other, indicating that different attrition rates could less parsimoniously explain the effect than a genuine change in goals. The same reasoning might apply to continuing-generation students, although the diminutions are somewhat more different. Still, given the cross-sectional nature of the present study, together with the fact that the number of observations in some cases is rather small (for third-year first-generation students), the present findings need to be replicated. Future research might employ a longitudinal design to more directly measure the evolution of students’ achievement goals. Alternatively, scholars might be willing to use publicly available large-scale data sets (e.g., National Longitudinal Survey of Youth, see Center for Human Resource Research [CHRR], 1994) to examine whether structural competition indeed moderates social class graduation gap.

Second, the use of different departments of the same academic institution as a proxy of competition creates a weakness for internal validity. Yet, it must be stressed that, in addition to structural differences in terms of selection, the Pilot Study showed that Life Sciences were indeed perceived as being more competitive than Civil Engineering. Such a difference was confirmed by the fact that third-year students enrolled in Life Sciences reported higher performance goals than those in Civil Engineering. However, one cannot exclude that the results could be due to a confounding variable (e.g., a field-specific academic socialization). Indeed, the present study should be considered as a single-site case study. Future research should manipulate competition in order to exclude possible confounds and draw causal conclusions.

## Conclusion

Adopting an even-handed approach (Duarte et al., 2014), this article reports preliminary evidence of a context-dependent effect of social class on mastery goals. On the one hand, first-generation students were argued to suffer from a particular lack of fit when enrolled in more competitive domains, which was found to prevent the maintenance of an optimal level of mastery goals. On the other hand, continuing-generation students were argued to suffer from a particular lack of fit when enrolled in less competitive domains, which was found—here also—to impair their mastery goals.

In other words, first-generation students—in addition to having lower degree aspiration (Zhang, 2005)—might be less likely to be learning-oriented and to persist when engaged in more competitive institution-driven upward mobility. Conversely, continuing-generation students—in addition to having higher degree aspiration (Chen and Carroll, 2005)—might be less likely to be learning-oriented and to persist when engaged in less competitive institution-driven (potential) downward mobility. A promising avenue for future scaled-up research would be testing whether these two complementary dynamics contribute to maintain the transmission of social inequalities from one generation to the next.

## Author Contributions

NS conceived and designed the study, collected the data, and analyzed it on the supervision of AQ and MJ. NS drafted the manuscript and AQ, MJ, and GM provided critical revisions. All authors approved the final version of the manuscript for submission.

### Conflict of Interest Statement

The authors declare that the research was conducted in the absence of any commercial or financial relationships that could be construed as a potential conflict of interest.

## Footnotes

1 Source: http://ogif.epfl.ch/

2 |SDR| refers to absolute Studentized Deleted Residuals.

3 http://www.ssp-sgp.ch/06_pdf/Code_deontologique.pdf

4 The seven choices were as follows: (i) less than compulsory school; (ii) compulsory school; (iii) apprenticeship; (iv) secondary school vocational diploma; (v) secondary school general diploma; (vi) advanced professional education; (vii) university; (vii) other (to specify in an open-ended question).

5 In this article we refer to mastery and performance goals in their approach form, as we aimed at focusing on the academically *adaptive* achievement goals (Kaplan and Flum, 2010). However, these goals might include an avoidance component and become more *maladaptive* (Murayama et al., 2012; for an illustration of the effects of social class on performance-avoidance goals, see Jury et al., 2015a). It is worth noting for the sake of transparency that three items assessing mastery-avoidance goals (e.g., “I worry that I may not learn all that I possibly could in classes”; α = 0.75, *M* = 4.31, *SD* = 1.33) and three performance-avoidance goals (e.g., “My goal in classes is to avoid performing poorly”; α = 0.67, *M* = 4.15, *SD* = 1.43) were added for exploratory purpose. We carried out the same analyses on mastery- and performance-avoidance goals. The results revealed only two significant effects, namely two main negative effects of academic years on the former, *B* = –0.91, *SE* = 0.16, *F*(1, 370) = 30.91, *p* < 0.001, and on the latter goals, *B* = –0.39, *SE* = 0.18, *F*(1, 370) = 4.54, *p* = 0.034. Both the importance of mastery- and performance-avoidance goals diminished in the third year compared to the first year.
